# Early differences in dynamic uptake of ^68^Ga-PSMA-11 in primary prostate cancer: A test-retest study

**DOI:** 10.1371/journal.pone.0246394

**Published:** 2021-02-02

**Authors:** J. olde Heuvel, B. J. de Wit-van der Veen, M. Sinaasappel, C. H. Slump, M. P. M. Stokkel

**Affiliations:** 1 Department of Nuclear Medicine, Netherlands Cancer Institute-Antoni van Leeuwenhoek, Amsterdam, The Netherlands; 2 Robotics and Mechatronics, Technical Medical Centre, University of Twente, The Netherlands; 3 Department of Medical Physics, Netherlands Cancer Institute-Antoni van Leeuwenhoek, Amsterdam, The Netherlands; IRCCS Ospedale Policlinico San Martino, Genova, ITALY

## Abstract

**Introduction:**

Dynamic PET/CT allows visualization of pharmacokinetics over the time, in contrast to static whole body PET/CT. The objective of this study was to assess ^68^Ga-PSMA-11 uptake in pathological lesions and benign tissue, within 30 minutes after injection in primary prostate cancer (PCa) patients in test-retest setting.

**Materials and methods:**

Five patients, with biopsy proven PCa, were scanned dynamically in list mode for 30 minutes on a digital PET/CT-scanner directly after an intravenous bolus injection of 100 MBq ^68^Ga-PSMA-11. Approximately 45 minutes after injection a static whole body scan was acquired, followed by a one bed position scan of the pelvic region. The scans were repeated approximately four weeks later, without any intervention in between. Semi-quantitative assessment was performed using regions-of-interest in the prostate tumor, bladder, gluteal muscle and iliac artery. Time-activity curves were extracted from the counts in these regions and the intra-patient variability between both scans was assessed.

**Results:**

The uptake of the iliac artery and gluteal muscle reached a plateau after 5 and 3 minutes, respectively. The population fell apart in two groups with respect to tumor uptake: in some patients the tumor uptake reached a plateau after 5 minutes, whereas in other patients the uptake kept increasing, which correlated with larger tumor volumes on PET/CT scan. Median intra-patient variation between both scans was 12.2% for artery, 9.7% for tumor, 32.7% for the bladder and 14.1% for the gluteal muscle.

**Conclusion:**

Dynamic ^68^Ga-PSMA-11 PET/CT scans, with a time interval of four weeks, are reproducible with a 10% variation in uptake in the primary prostate tumor. An uptake plateau was reached for the iliac artery and gluteal muscle within 5 minutes post-injection. A larger tumor volume seems to be related to continued tumor uptake. This information might be relevant for both response monitoring and PSMA-based radionuclide therapies.

## Introduction

PET/CT imaging is increasingly used for targeted diagnostic imaging in prostate cancer (PCa), since radiopharmaceuticals labeled to the Prostate-specific Membrane Antigen (PSMA) were introduced 5 years ago [1–6]. The first studies with PSMA-labeled radiotracers from the group of Haberkorn and Eder mainly focused on its’ clinical applicability in recurrent PCa. These studies suggested that a 60 minutes tracer uptake is optimal for good visual tumor-to-background contrast in ^68^Ga-PSMA-11 PET/CT imaging [7–9]. The current EANM/SNMMI procedure guideline for PSMA-PET/CT also adapted this uptake time (acceptable range 50–100 minutes), with the remark that late imaging up to 3-4h after injection may increase sensitivity for lesion detection. Though uptake-times longer than 100 minutes have reduced count rate (especially relevant for ^68^Ga), late imaging may help better identify lesions close to ureters or in patients with low PSMA-expression or PSA levels [9, 10].

Nowadays, PSMA-PET/CT not only plays an eminent role in localizing recurrent PCa [11], but also in the diagnosis and staging of primary PCa, especially for high risk patients [12]. Intense bladder accumulation present 45–60 minutes post-injection is known to hamper the visual assessment of the primary tumor, especially on the basal side of the prostate [13, 14]. Furosemide can be administered after PSMA-ligand injection to increase renal clearance, and hence, reduce tracer accumulation in the urinary system. Early time-point imaging of the pelvic area on the other hand may also reduce the effects of urine accumulation. Multiple studies have reported on dynamic or multi time-point ^68^Ga-PSMA imaging in primary and recurrent PCa, to evaluate the effectiveness in primary PCa (see S1 Table for details) [9, 10, 15–20]. In this setting, ‘true’ dynamic PET/CT (dPET/CT) refers to visualization of a limited field of view (FoV) with high temporal sampling (typical <5 minutes), while multi time-point imaging revers to static acquisitions of a larger FoV with a lower temporal sampling (typical >5 minutes). Both imaging approaches provide temporal data which may also hold valuable biological information for lesion characterization.

Yet, limited intra-prostatic imaging data is currently available regarding the distribution phase of ^68^Ga-PSMA-11 (e.g., the first 30 minutes post-injection). The repeatability of visual aspects and quantitative measures describing both the temporal uptake profile and spatial intra-prostatic distribution pattern are currently unknown, but is relevant when applying early time-point PET for visualization of the basal prostate. Additionally, this information is needed for our research regarding intraoperative ^68^Ga-PSMA Cerenkov imaging, where early dynamics might gain insight in the timing of intraoperative injection (NL8256). Next, if considered repeatable, pre-operative PET imaging can be used as guidance for intraoperative injection [21]. So, the objective of this study was to assess intra-prostatic ^68^Ga-PSMA-11 uptake during the first 30 minutes after injection in patients with primary PCa in test-retest setting.

## Materials and methods

### Study population

This prospective observational cohort study was approved by the Antoni van Leeuwenhoek Medical Ethics Committee (NL8263) and all patients signed written informed consent. The study protocol can be found in S1 File. No sample size calculation was performed, as the study has only a explorative and descriptive nature, additionally this dynamic imaging sub study was part of a larger clinical trial. Patients were selected at the multidisciplinary team meeting between December 2018 and February 2019 in the Netherlands Cancer Institute—Antoni van Leeuwenhoek. Eligible patients were aged 18 years or older, had histologically confirmed PCa (biopsy proven) and fulfilled one of the three local criteria for the ^68^Ga-PSMA-11 PET/CT-scan (≥cT3, Gleason score ≥4+3 = 7, or PSA ≥20 ng/mL). Six patients were recruited by nurse practitioners or the researcher (JoH) and the study took place in the Netherlands Cancer Institute—Antoni van Leeuwenhoek. Patients were excluded if no ^68^Ga-PSMA expression was visible on the first PET-scan or when treatment was started in between the two scans.

### PET/CT image acquisition

Both dynamic and static ^68^Ga-PSMA-11 PET/CT-scans were acquired twice on a digital PET/CT system (Vereos, Philips, Best, the Netherlands) with a four-weeks interval. The ^68^Ga-Glu-urea-Lys(Ahx)-HBED-CC (^68^Ga-PSMA-11) (Scintomics, Fürstenfeldbruck, Germany) was produced as described previously [22].

Patients were not required to fast prior to the scans, and in contrast to EANM guidelines, no furosemide was administered prior to dynamic imaging so not to induce the urge to void during the scan. A dPET/CT-scan was acquired in list mode 30 seconds before an intravenous bolus injection (10mL) of 100 MBq of ^68^Ga-PSMA-11 according to local clinical protocol, until 30 minutes post-injection (p.i.). The acquisition was performed from one bed position (16.4cm) with the prostate in the central FoV. A low-dose CT-scan (with 120 kV, 30mAs with dose modulation, spiral acquisition, reconstruction in 2 mm slices, and scan range from proximal femora to iliac crest) was made before tracer injection for attenuation correction and anatomic localization [15]. Dynamic data was binned into 30 frames of 1 minute for quantitative evaluation and into 6 frames of 5 minutes for visual assessment. OSEM image reconstruction was performed with isotropic voxel size of 4 mm at 3 iterations and 15 subsets without filters or PSF correction, while applying all usual corrections such as for decay, scatter, attenuation, randoms and dead-time.

Subsequently, a static whole body PET/CT scan was perfomed according to local clinical protocol 45 minutes p.i. and reconstructed with voxel size of 4 mm (3 iterations, 15 subsets). CT settings were equal to the dynamic settings, but the scan range was from proximal femora to skull base. Subsequently, an additional acquisition of one bed position around the prostate was acquired (~70 minutes p.i.). The second PET/CTacquisition session was scheduled four weeks later according to the same protocol with an allowed deviation of <10% in administered activity.

### Image analysis

Semi-quantitative evaluation of the dPET data was performed with MATLAB R2017b (The MathWorks, Natick, US, 2017). Regions of interest (ROIs) were manually drawn in the prostate tumor, bladder, gluteal muscle and common iliac artery, and should have a diameter of at least 15 mm (1.8cm^3^) to reduce partial volume effects. The prostate tumor was manually segmented, based on the location and lesion size as displayed on the T2-weighted MRI scan. The arterial ROI (diameter ~10 mm) was placed in the first frame over 5 consecutive slices and propagated to the subsequent frames. The ROIs for the tumor and gluteal muscle were placed in the last time frame and back propagated to prior frames. Time-activity curves (TACs) were generated for the entire dynamic time range. An image derived input function from the common iliac artery was used to describe the distribution of ^68^Ga-PSMA in the blood pool, as described in [23]. For the second dPET/CT, all ROIs were redefined and not copied from the previous scan. The volume of the prostate lesion was assessed on the static PSMA PET/CT-scan, using 3D slicer v4.10.0 (slicer.org) to segment the tumor using the PET segmentation extension. This is based on the optimal surface segmentation approach [24], and the segmentation could be manually refined, again also keeping in mind the location and size of the lesion on MRI.

Tracer uptake was defined as either lean body mass (LBM) corrected standardized uptake value (SUL_mean_) or tumor-to-blood ratio (TBR). The intra-patient variation was expressed as percentage change and the inter-patient variation is expressed as the Coefficient of Variation (CoV). An uptake ‘plateau’ was defined as the time where the slope of the uptake is close to zero with a maximum of 0.2. The start of bladder filling was defined as the time where the uptake in the bladder is twice the uptake of the gluteal muscle (background).

## Results

Six patients were enrolled in this prospective observational cohort study. One patient was excluded for analysis, since this patient had a PSMA-negative tumor. The median time between two scans was 24 days (range 22–28 days). The median net injected activity was 105.9 MBq (range 80.7–108.4 MBq) for the first and 102.5 MBq (range 96.23–106.46MBq) for the second scan. Further demographics are shown in Table 1.

**Table 1:** Demographics of the 5 patients included in this study. Data is shown as absolute number (percentage) or as mean (interval).

Patient	1	2	3	4	5
**Age (years)**	70	73	67	73	76
**Gleason score biopsy**	5+4 = 9	3+4 = 7	4+3 = 7	4+3 = 7	4+3 = 7
**Gleason score post RALP**	5+4 = 9	4+4 = 8	x	3+4 = 7	4+3 = 7
**iPSA (ng/mL)**	6.5	8.2	60	6.9	16.4
**TNM**	cT2N0M0	cT3bN0M0	cT3aN1M0	cT1cN0M0	cT1cN0M0
**Prostate volume on MRI (cc)**	70	69	50	54	46
**Lesion size on MRI (mm)**	17×8	34×44×28 (L)	41×15×41	9×5×5	Whole prostate
		11×11 (S)			
**Dosage scan 1/scan 2 (MBq)**	106.5/83.2	108.4/105.4	98.8/80.7	105.1/96.2	105.3/102.5
**Lesion size on PET (cc)**	4	31 (L)	48	2	30
		2 (S)			
**Tumor-blood ratio on PET**	2.0	9.1	10.8	3.09	6.3
**Therapy**	RALP	RALP	exRT	RALP	RALP
**PSMA accumulation (stable or rising)**	Stable	Rising	Rising	Stable	Rising
**ADC value on MRI (mm**^**2**^**/s)**	1076	793 (L)	720	826	1121
**b-value (0-200-800)**		886 (S)			

iPSA–Prostate specific antigen, RALP–Robotic assisted Laparoscopic Prostatectomy, exRT–external radiotherapy. Note that patient 2 has a large (L) and a small (S) lesion.

### Repeatability of ^68^Ga-PSMA accumulation

**Fig 1:**
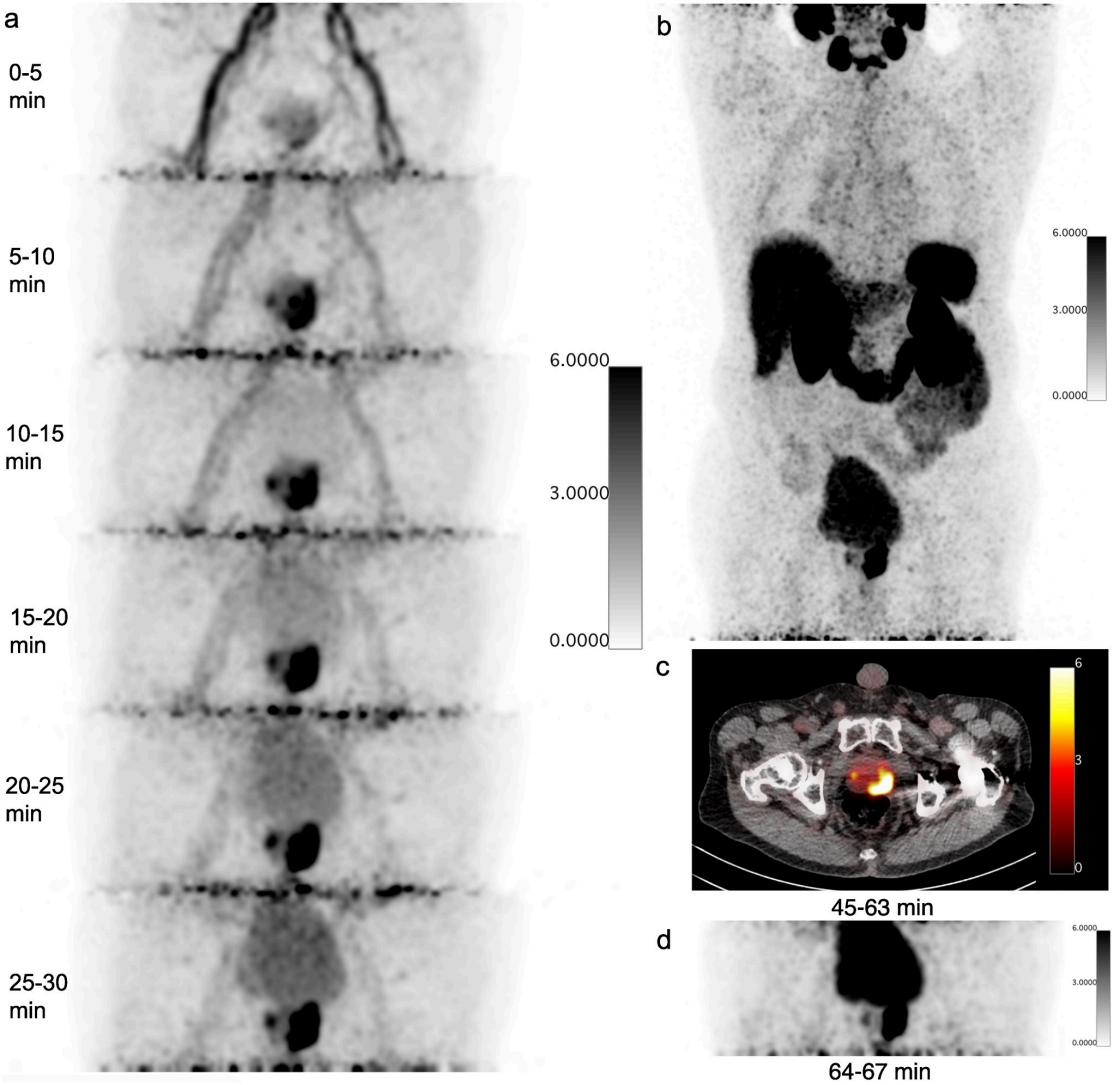
A typical example of dynamic and static PSMA uptake (patient 2). A) The dynamic PET-data is reconstructed into a time frame of 5 minutes. One can tendentious observe the prostate tumor uptake in minute 5–10. In the last time frame, the bladder activity becomes conspicuous. B) Maximum Intensity Profile of the regular clinical static scan in shown. C) PET/CT fusion of the static scan in transverse direction. In D) the delayed static scan of the pelvic region is shown.

**Fig 2:**
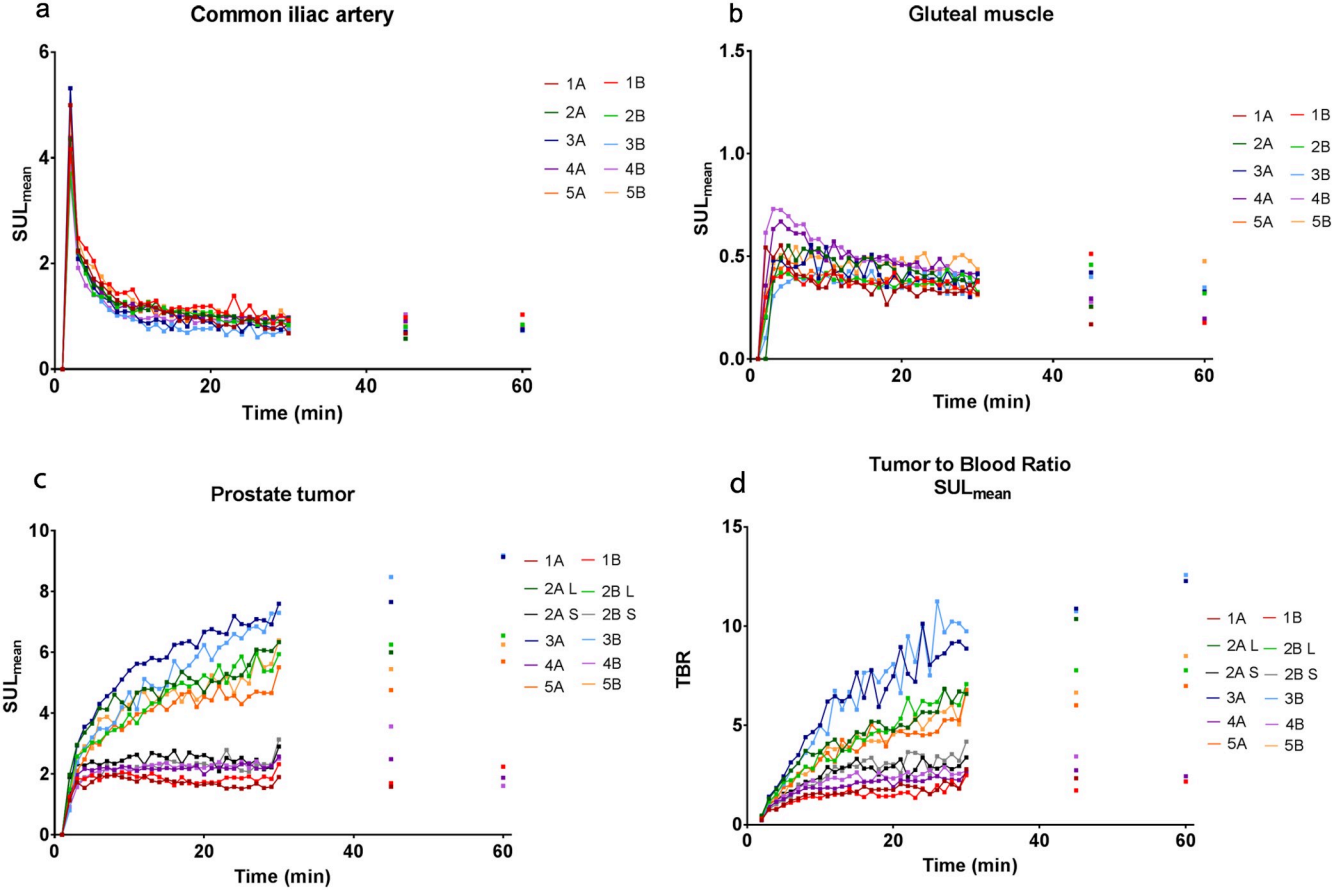
The SUL_mean_ time activity curve of all patients for the common iliac artery (A), gluteal muscle (B), prostate tumor (C), and the TBR (D). The TAC of the prostate tumor shows clear deviation in patients 1. 2S,4 which become stable in uptake after 5 minutes and the 2L,3,5 which keep increasing. Note that patient 2 has a large (L) and a small (S) lesion.

A typical example of ^68^Ga-PSMA-11 accumulation over time in the pelvic region is presented in Fig 1. Evaluation of the dynamic data shows tumor accumulation already within the first 5 minutes in all patients, while mean±SD bladder filling was seen at 9±5 minutes p.i. (see TACs in Fig 2). The arterial blood input curve demonstrates a steep inflow phase with highest SUL_mean_ in the first minute (SUL_mean_ 4.3 [range: 3.6–5.4]), whereas after 5±1 minutes a plateau was reached in all patients (SUL_mean_ 1.2 [0.8–1.8]). In all patients, the uptake plateau in the gluteal muscle was reached after 3±1min. Continuing accumulation in the prostate tumor was observed in two patients for the full duration (30 minutes) of the scan, while in two patients tumor accumulation plateaued after 5±1min. One patient (no. 2) had two lesions, one which showed increased uptake and one who plateaued (Figs 1 and 2).

^68^Ga-PSMA-11 uptake at the second dPET/CT-scan followed the same uptake pattern in the prostate tumor, gluteal muscle and common iliac artery (Fig 2). The intra- and interpatient variation in uptake is provided in Table 2. The largest intra patient variation was observed in bladder TAC (median 32.7%), while tumor (9.7%), gluteal (14.1%) and arterial accumulation (12.2%) proved to be repeatable over time.

**Table 2:** Inter- and intra-patient variability in SUL_mean_ of the dynamic and static PET. Data is shown as median (range).

	Dynamic PET			Whole body static PET		
Site	SUL_mean_^a^	Intra-patient (%)	Inter-patient CoV (%)	SUL_mean_^a^	Intra-patient (%)	Inter-patient CoV (%)
**Artery**	4.3 (3.6–5.3)	12.2 (3.4–13.8)	12.5	0.8 (0.6/1.0)	14.1 (3.5–45)	17.1
**Tumor**	5.7 (1.9/7.6)	9.7 (1.1–16.5)	40.5	5.1 (1.5/8.5)	10.7 (4.4–43.1)	50.3
**Muscle**	0.4 (0.3/0.5)	14.1 (3.0–18.1)	27.0	0.3 (0.2/0.5)	13.2 (5.8–22.9)	32.8
**Bladder**	8.4 (1.5/20.2)	32.7 (11.8–100.4)	94.1	6.4 (2.1/26.8)	47.3 (5.0–118.8)	90.4

^a^The highest value for SUL_mean_ on the PET is provided here.

### Tumor PSMA accumulation

As mentioned before, not all patients showed an increase in tumor accumulation for the entire duration of the scan. The six lesions of the five patients can be divided into two groups based on the TACs after 5 minutes: one group with stable uptake after 5 minutes (n = 3) and the other with an ongoing increase in uptake after 5 minutes (n = 3). Note that one patient had two intra-prostatic lesions both with a different uptake pattern. Looking at the first 5 minutes (absorption phase), both groups follow the same pattern. At this absorption phase the SUL varies between the groups, though nog significant (3.5 versus 2.1, *p*-value 0.1), while the SUL at 20 minutes post-injection differs significantly (5.4 versus 2.1, *p*-value 0.003). These patterns were visible both in the test and retest setting.

**Fig 3:**
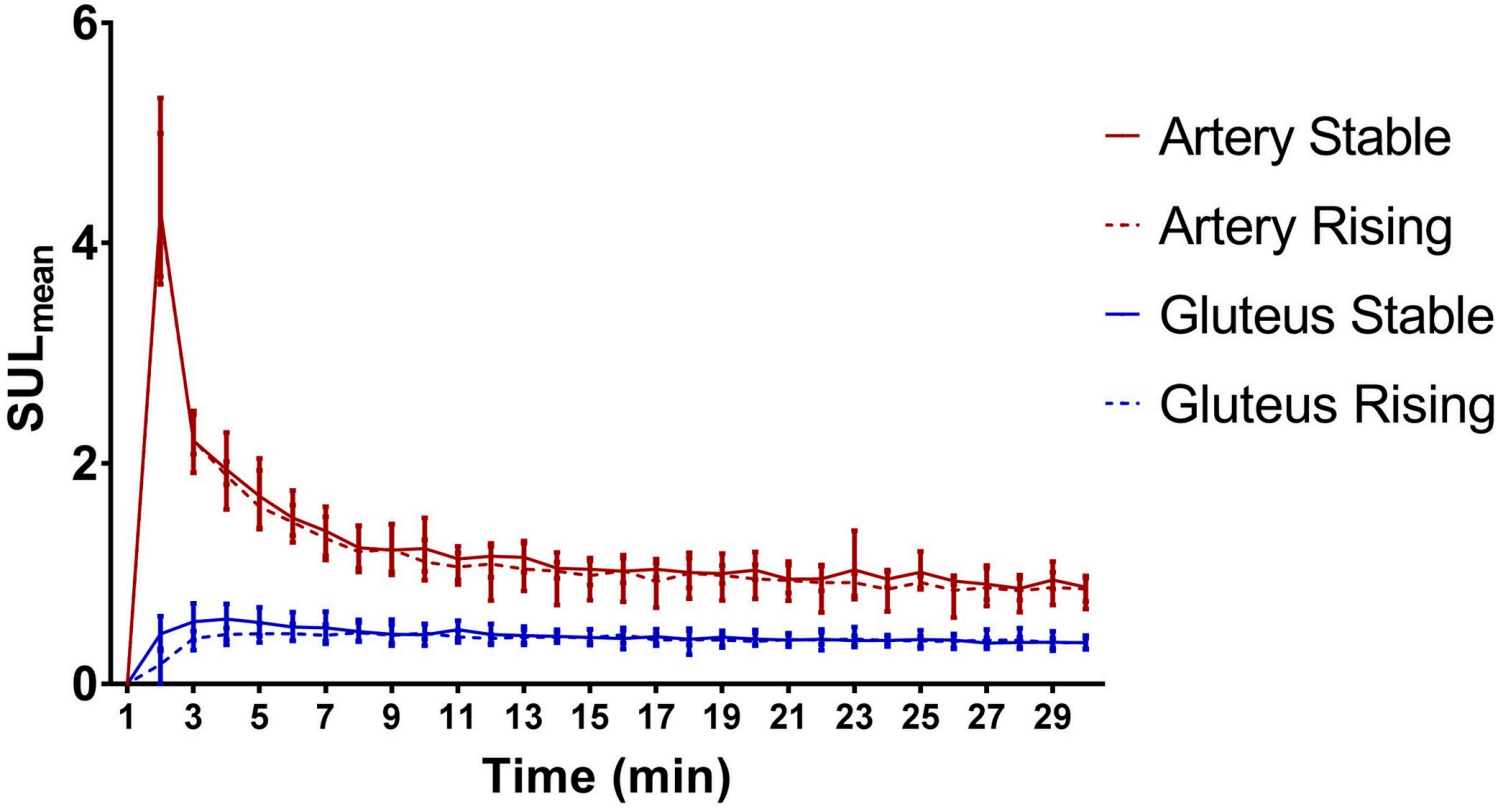
Average time activity curve (±range) of the artery and gluteal muscle, separated into two groups with different uptake patterns in the tumour, in blue the stable group and in red the rising group.

No evident difference in demographics (Gleason score or initial PSA) was observed between the two groups (Table 1). In addition, also no differences between arterial and gluteal muscle uptake patterns (Fig 3) or tissue diffusion as measured on ADC-MRI was present. The only evident difference was tumor volume, which is about 12 times higher in patients that kept rising (36cc vs 3cc). This difference also occurred in a patient with two different uptake patterns within one scan, with a large and small lesion (31 cc vs 2cc).

## Discussion

In the present study, we have evaluated the intra-prostatic ^68^Ga-PSMA-11 uptake during the first 30 minutes after injection in patients with primary PCa in test-retest setting. The goal was to gain insight into the dynamic behavior and its repeatability related to intra-operative CLI imaging [21]. The intra patient variation of the TACs was below 15% for the artery, tumor and gluteal muscle, showing the repeatability of dynamic imaging in primary prostate cancer. The variation of the bladder was higher (32.7%), which is in line with the expectations based on urinary excretion. This finding is consistent with the repeatability of whole body static PSMA PET/CT scans 45–60 minutes p.i. [25, 26].

With respect to tumor accumulation, this was already visible within the first 5 minutes p.i., which is consistent with the studies by Kabasakal *et al*. [16], Uprimny *et al*. [15] and Schmuck *et al*. [17]. The latter two studies, showed comparable TACs of the gluteal muscle and artery as found in our study, with stable uptake after 5 and 3 minutes respectively. Early imaging might enable better visualization of the prostate tumor, especially at the basal side, as intense urine accumulation in the bladder was seen at 9±5 minutes p.i. In contrast, some studies promote late PSMA-PET scans [10, 17], which can be especially beneficial for lymph node detection. However, imaging four hours p.i. means that the PET-signal is lower and effects of image noise are higher. Thus, SUL values in late time-point scans should be carefully interpreted. Though early and late time point imaging both have their pros and cons, there is no clear difference in the number of lesions detected between the two approaches [16, 17]. So, regarding intra-operative CLI imaging with ^68^Ga-PSMA-11 it was concluded that test-retest accumulation patterns are highly repeatable, and intra-operative detection can commence as early as 5 minutes p.i.

### Stable and rising uptake in PCa

Visualization of the individual prostate tumor TACs revealed a distinct difference between the patients. Three lesions showed a stable uptake profile after five minutes, whereas three lesions had increasing tumor uptake over time. This effect is not yet described in literature, we think, mainly because previous literature reported the mean TAC of all patients, thus obscuring this effect. The difference in TAC shape might be explained by multiple causes, such as systemic distribution of ^68^Ga PSMA-11, tumor biology, technical differences and tumor perfusion, which each will be discussed below.

After intravenous injection of ^68^Ga-PSMA-11, it is distributed proportionally through all tissues by the systemic blood flow, so a reduced tumor accumulation might be explained by a limited systemic supply of ^68^Ga-PSMA-11. When comparing the uptake in the common iliac artery and gluteal muscle, there was no clear difference between both patient groups (arterial SUL_mean (t = 2min)_ stable:4.3 vs. rising:4.2; gluteal SUL_mean (t = 30min)_ stable:0.4 vs. rising:0.4). Therefore, it is unlikely that the difference in tumor uptake is explained by a reduced systemic supply of ^68^Ga-PSMA-11.

Technical differences, like reconstruction parameters or tracer activity and uptake time, are also known to influence the quantitative values especially in smaller lesions. However, present patients were scanned on a the same scanner, with equal imaging and reconstruction protocols. Furthermore, the measures of each individual patient were repeatable as shown in Table 2. Since tumor volume turned out to be the only relevant parameter, the partial volume effect might explain the difference in uptake [27]. In quantitative PET-imaging, the recovery coefficient is lower for smaller lesions [28], thus implying that the measured uptake is proportional to lesion size. Still, in the current study, the lesion volume did not change over time resulting in a constant underestimation for small lesions, thus not explaining the different shapes of the TACs between the groups.

Perfusion, defined as the rate in which blood flows through the vascular bed of a tissue, can induce heterogeneous accumulation patterns. In large tumors one can presume that not the entire tumor is adequately supplied, despite the upregulated angiogenesis and increased permeability of vessels [29, 30]. With dynamic contrast-enhanced MRI perfusion and vascular permeability can be assessed [31], so in future studies this might be considered.

The actual tumor phenotype might also be a factor to clarify the different uptake curves. Since ^68^Ga-PSMA-11 binds to the PSMA-receptor, the difference might be caused by variations in expression. A plateau in the TAC graphs might indicate total occupancy of the receptors by ^68^Ga-PSMA suggesting that the number of receptors is low in smaller sized tumors. In bigger tumors, on the other hand, there is such a large amount of receptors that the saturated state will not be accomplished within the scanning time of 30 minutes. To test this hypothesis, one should look at the receptor immunohistochemistry staining that matches with the radiolabeled PSMA-ligand. Woythal *et al*. found that a high SUV_max_ 60 minutes post-injection correlates to a tumor lesion with a high immunoreactive score. Still, this does not mean by definition a bigger lesion, as they did not find a correlation between the size of the lesion and SUV_max_ [32]. If indeed a selection of patients show saturation after administration of the ligand, this observation will be relevant for both response monitoring and PSMA-based radionuclide therapies. The amount of PSMA peptide in ^68^Ga-PSMA-11 is typically lower than10μg, and for Lutetium-177 PSMA this is much higher 250μg per cycle. So, for diagnostic imaging this induces a dependency of SUV-measures from ligand concentration and tumor load, whereas for peptide therapies it may result in overdosing of patients with limited tumor load. However, the current population is small, and more research is needed to confirm these findings.

## Limitations

The current study has some limitations that need to be addressed. First of all, the number of patients is too small to draw definite conclusions, even though the differences in absorption phase and tumor SUL_t = 20min_ between the groups seem significant. Still, we think our findings demonstrate an interesting phenomenon, which is worth exploring in the future. Second, the TACs of the first 30 minutes were not continued to the two later static time points, since extrapolating the curve from this single time point will enter too much uncertainty about the uptake pattern. Nevertheless, those standalone points gave valuable information, since tumor uptake was divided in the same groups. As there is still no consensus or standardization in segmentation of primary prostate cancer lesions on PSMA PET/CT, manual segmentation was used in this study instead of a threshold-based segmentation. Though generally not preferred, a study by Zamboglou showed that manual segmentation outperformed the fixed threshold method in PSMA-PET, when comparing the size of the segmentation to the histopathological size of the lesion [33]. Still, accumulation on PET is inherently prone to partial volume effects and only provides average accumulation over relatively large voxels, thus thereby limiting the ability to assess intra-tumor heterogeneity and multifocality. Next, in this study we did not perform arterial blood sampling, to verify plasma tracer input functions. However, image-based tumor-to-blood ratios were validated as a simplified method to quantify uptake [34] as shown in Fig 2.

## Conclusion

Dynamic ^68^Ga-PSMA-11 PET/CT-scans are reproducible within a 4 week time period. In general, 5 minutes post injection a plateau is reached for the artery, gluteal muscle and for some prostate tumors. There seems to be a deviation after 5 minutes in patients with a large tumor volume where uptake increases over time and stable uptake in patients with a small tumor volume. This information might be beneficial for patient selection for radionuclide therapy with PSMA as target and early time-point imaging.

## Supporting information

S1 Table Overview of published dynamic studies with PSMA PET/CT. (PDF)

S1 File Study protocol. (PDF)

S2 File TREND statement checklist. (PDF)
