# Supplementary material for: Early differences in dynamic uptake of 68Ga-PSMA-11 in primary prostate cancer: A test-retest study
Source: PLoS One. 2021 Feb 2;16(2):e0246394. doi: 10.1371/journal.pone.0246394 (PMC7853454; doi:10.1371/journal.pone.0246394)
Supplement: S1 Table — (PDF) [file pone.0246394.s001.pdf]

Supplementary Table 1. Overview of published dynamic studies with PSMA PET/CT.

Author	Uprimny [15]	Afshar-oromieh [10]	Kabasakal [16]	Schmuck[17]	Sachpekidis [18]	Afsfar-oromieh [9]	Sachpekidis [20]	Sachpekidis [19]
Year	2016	2017	2015	2017	2016	2016	2016	2018
No. Patients	55	112	28	20	24	4	31	16
Primary or recurrent cancer patients	Both	Both	Both	Primary	Primary	Recurrent	BCR	Local recurrence
Isotope	^{68}Ga-PSMA-11	^{68}Ga-PSMA-11	^{68}Ga-PSMA HBEC	^{68}Ga-PMSA I&T	^{68}Ga-PSMA-11	^{68}Ga-PSMA-11	^{68}Ga-PSMA-11	^{68}Ga-PSMA-11
Dynamic scanning	Yes	No, 2 time points	No, 2 time point	Yes, and 2 time points	Yes and static	6 time points	Yes and static	Yes and static
Time	8min	1h & 3h	5min 45/60min	10 min, 1h & 3h	60 min	5 min, 1,2,3,4&5 h	60 min	60 min
FoV	15.6cm	WB/pelvis only	WB	WB	44cm	WB	44cm	44cm
Injected Activity (MBq)	150	207	113	102	268	173		
Dynamic Acquisition protocol	60s per frame	Not performed	NS	12 frames ×10s, 4×30s, 2×180s	10 frames ×30s, 5×60s, 5×120s, 8×300s	Not performed	10 frames ×30s, 5×60s, 5×120s, 8×300s	10 frames ×30s, 5×60s, 5×120s, 8×300s
Static Acquisition protocol	Not performed	0.7cm/min	3min/bed	0.7mm/s	2min/bed	3min/bed	2min/bed	2min/bed
Quantitative measures	SUV_{max}	SUV_{max} SUV_{mean}	SUV_{max}	SUV_{peak} SUV_{mean}	SUV_{max} SUV_{mean}	SUV_{max} SUV_{mean}	SUV_{max} SUV_{mean}	SUV_{max} SUV_{mean}
Furosemide given	NS	Yes	NS	Yes, after dynamic	NS	NS	NS	NS
Reconstruction algorithm	OSEM 4i8ss	2i21ss G5mm	NS	2i22ssG5mm	OSEM 6i12ss	OSEM 2i8ssG2mm	OSEM 6i12ss	OSEM 6i12ss
Software used	Hermes	NS	NS	PMOD	PMOD	PMOD	PMOD	PMOD
Tumor segmentation method	Manual	70% SUL_{max}	Manual	40% SUL_{max}	Pseudo snake isocontour	70% SUL_{max}	Pseudo snake isocontour	Pseudo snake isocontour

NS: Not specified, WB: Whole body, G: Gaussian, i: iteration, ss: subsets, OSEM: ordered subset expectation maximization, FoV: Field of View
