# Supplementary material for: Early differences in dynamic uptake of 68Ga-PSMA-11 in primary prostate cancer: A test-retest study
Source: PLoS One. 2021 Feb 2;16(2):e0246394. doi: 10.1371/journal.pone.0246394 (PMC7853454; doi:10.1371/journal.pone.0246394)
Supplement: S1 File — (PDF) [file pone.0246394.s002.pdf]

Variation on PSMA Receptor Expression over time in prostate cancer

Version number:2.0, 05-07-2018

PROTOCOL TITLE ‘Variation on PSMA Receptor Expression over Time in prostate cancer’

**Protocol ID**	**PRET**
**Short title**	**PSMA Receptor Expression over Time (PRET)**
**Version**	**2.0**
**Date**	**05-07-2018**
**Coordinating investigator/project leader**	**M.P.M. Stokkel, MD, PhD**<br>**Dept. of Nuclear Medicine**<br>**Antoni van Leeuwenhoek Hospital – Netherlands Cancer Institute.**
**Principal investigator(s) (in Dutch: hoofdonderzoeker/ uitvoerder)**	**J. olde Heuvel, MSc**<br>**PhD-candidate**<br>**Dept. of Nuclear Medicine**<br>**Antoni van Leeuwenhoek Hospital – Netherlands Cancer Institute.**
**Subsidising party**	**Koningin Wilhelmina Fonds voor de Nederlandse Kankerbestrijding- Stichting voor de Technische Wetenschappen KWF-STW project grand number 15175**

TABLE OF CONTENTS

1. INTRODUCTION AND RATIONALE 6
2. OBJECTIVES 7
3. STUDY DESIGN 7
3.1 PSMA expression over time 7
4. STUDY POPULATION 8
4.1 Population (base) 8
4.2 Inclusion criteria 8
4.3 Exclusion criteria 8
4.4 Sample size calculation 8
5. NON-INVESTIGATIONAL PRODUCT 9
5.1 Name and description of non-investigational product(s) 9
5.2 Summary of findings from non-clinical studies 9
5.3 Summary of findings from clinical studies 9
5.4 Summary of known and potential risks and benefits 9
5.5 Description and justification of route of administration and dosage 9
5.6 Dosages, dosage modifications and method of administration 9
5.7 Preparation and labelling of Non-Investigational Medicinal Product 9
5.8 Drug accountability 9
6. METHODS 10
6.1 Study parameters/endpoints 10
6.2 Study procedures 10
6.3 Withdrawal of individual subjects 10
6.4 Replacement of individual subjects after withdrawal 10
6.5 Follow-up of subjects withdrawn from treatment 11
6.6 Premature termination of the study 11
7. STATISTICAL ANALYSIS 12
7.1 Primary study parameter(s) 12
7.2 Secondary study parameter(s) 12
7.3 Interim analysis (if applicable) 12
8. ETHICAL CONSIDERATIONS 13
8.1 Regulation statement 13
8.2 Recruitment and consent 13
8.3 Objection by minors or incapacitated subjects (if applicable) 13
8.4 Benefits and risks assessment, group relatedness 13
8.5 Compensation for injury 13
9. ADMINISTRATIVE ASPECTS, MONITORING AND PUBLICATION 14
9.1 Handling and storage of data and documents 14
10. STRUCTURED RISK ANALYSIS 15
10.1 Potential issues of concern 15
10.2 Synthesis 15
11. REFERENCES 16

LIST OF ABBREVIATIONS AND RELEVANT DEFINITIONS

^{18}F-FDG	2-[F-18]-fluoro-2-deoxy-D-glucose
^{18}Ga-PSMA	Gallium-68-Prostate Specific Membrane Antigen
ABR	ABR form, General Assessment and Registration form, is the application form that is required for submission to the accredited Ethics Committee (In Dutch, ABR = Algemene Beoordeling en Registratie)
AE	Adverse Event
AR	Adverse Reaction
CA	Competent Authority
CT	Computed Tomography
CCMO	Central Committee on Research Involving Human Subjects; in Dutch: Centrale Commissie Mensgebonden Onderzoek
CV	Curriculum Vitae
DSMB	Data Safety Monitoring Board
dPET/CT	Dynamic PET/CT
EU	European Union
GCP	Good Clinical Practice
IB	Investigator’s Brochure
IC	Informed Consent
METC	Medical Research Ethics Committee (MREC); in Dutch: Medisch Ethische Toetsing Commissie (METC)
NKI-AVL	Netherlands Cancer Institute – Antoni van Leeuwenhoek
PCa	Prostate Cancer
PET	Positron Emission Tomography
PSMA	Prostate Specific Membrane Antigen
Sponsor	The sponsor is the party that commissions the organisation or performance of the research, for example a pharmaceutical company, academic hospital, scientific organisation or investigator. A party that provides funding for a study but does not commission it is not regarded as the sponsor, but referred to as a subsidising party.
SUSAR	Suspected Unexpected Serious Adverse Reaction
Wbp	Personal Data Protection Act (in Dutch: Wet Bescherming Persoonsgevens)
WMO	Medical Research Involving Human Subjects Act (in Dutch: Wet Medisch-wetenschappelijk Onderzoek met Mensen

SUMMARY

Rationale: Prostate cancer is the third most common cancer in Europe and is still the leading cancer among men in the Netherlands with over 13,000 men diagnosed each year. 68-Gallium Prostate Specific Membrane Antigen (^{68}Ga-PSMA) is an emerging imaging agent that already has been widely proven as a highly effective agent for restaging PCa cells, but also holds enormous potential for initial staging and image guided surgery. During surgery, ^{68}Ga-PSMA can potentially be used for the assessment of resection margins based on Cerenkov Light emission. The interval between initial staging and subsequent prostatectomy surgery, however, ranges from 4 to 6 weeks. Since little is known about the PSMA expression over time without intervention, a study on this is required. If PSMA expression is equal between both scans, the inclusion and dose determination for image guided surgery can be based on the clinical ^{68}Ga-PSMA PET/CT scan. In order to validate this, two PET scans will be performed in a test-retest setting in primary prostate cancer patients. Dynamic imaging will be performed in order to get insight into the optimal scan time. Subsequently this will be used also to establish the ideal timing of surgery after injection.

Objective: The objective of this study is to obtain preliminary data on variability in PSMA expression by comparing the tracer uptake on conventional clinical ^{68}Ga-PSMA PET/CT with an additional ^{68}Ga-PSMA PET/CT (4 weeks later) within primary prostate cancer patients. Dynamic imaging will be performed in order to get insight into the most ideal scan time.

Study design: This is a prospective observational study.

Study population: This study will include 30 primary prostate cancer patients.

Intervention (if applicable): Not applicable.

Main study parameters/endpoints: The main study endpoint is to evaluate the PSMA expression variation at 4 week intervals. The secondary endpoint is to determine the optimal time between ^{68}Ga-PSMA injection and prostate surgery for future image-guided surgery.

Nature and extent of the burden and risks associated with participation, benefit and group relatedness: The patients included in this study have an additional radiation exposure of 6.3mSv, since they need to undergo an additional ^{68}Ga-PSMA PET/CT-scan. This is 9.3mSv for the patients with the dynamic PET/CT-scan.

1. INTRODUCTION AND RATIONALE

Prostate cancer (PCa) is the second most common cancer amongst men in the world, recorded in 2012[1]. And the most common among men in the Netherlands[2].

Positron Emission Tomography (PET) / Computed Tomography (CT) scanners are used to detect malignancies throughout the body. For PCa 68-Gallium Prostate Specific Membrane Antigen (^{68}Ga-PSMA) is a tumour specific radiopharmaceutical. Since PSMA is a membrane-bound enzyme which is overexpressed in PCa cells in comparison to benign prostatic tissue (approximately 100- to 1000-fold more PSMA expression)[3]. This makes ^{68}Ga-PSMA an emerging imaging agent that already has been widely proven as a highly effective detector of PCa cells, and therefore, holds enormous potential for medical imaging[4]. In the last years, ^{68}Ga-labelled tracers are increasingly used for PET-imaging due to the in-house availability of GMP-approved ^{68}Ga generators. Nowadays, ^{68}Ga-based tracers are commonly used within Europe for targeted diagnostic imaging in specific tumour types like prostate cancer[5–8]

Three large-scale European studies reported detection rates for recurrent prostate cancer above 95%[9–11]. Afshar-Oromieh et al.[9] showed positive and negative predictive values of, respectively, 91.4% and 100% in 319 prostate cancer patients. Eiber et al.[10] found a 96.8% detection rate in 248 patients with biochemical recurrence after radical prostatectomy using PET/CT. Maurer et al.[11] reported a 95.2% accuracy in 130 patients with intermediate to high-risk prostate cancer. The same study found that ^{68}Ga-PSMA was significantly superior to conventional anatomical imaging ($p<0.001$). Rahbar et al.[12] found a very high agreement between ^{68}Ga-PSMA and histopathology. Due to the superiority of ^{68}Ga-PSMA over other imaging approaches, ^{68}Ga-PSMA is fast emerging as the gold standard for *in vivo* detection of prostate cancer[13]. The PET signal depends on the number of cells expressing the membrane enzyme, PSMA expression per cell and the imaging system resolution. In general the tumour uptake correlates with the absolute cell surface PSMA expression. [14]

^{68}Ga-PSMA is an emerging imaging agent that already has been widely proven as a highly effective agent for restaging PCa cells, but also holds enormous potential for initial staging and image guided surgery. During surgery, ^{68}Ga-PSMA can be probably be used of assessment of the resection margins based on Cerenkov Light emission. This is part of study granted by KWF (grant number 15175). The interval between initial staging and its application in surgical setting, however, ranges from 4 to 6 weeks. Since little is known about the PSMA expression over time without intervention, a study on this is required to verify this. If PSMA expression is equal between both scans, the inclusion and dose determination can be based on the clinical PET/CT scan. In order to validate this, two PET scans will be performed in a test-retest setting in primary prostate patients. Dynamic imaging will be performed in order to get insight into the most ideal scan time. Subsequently this will be used also to establish the ideal timing of surgery after injection.

2. OBJECTIVES

Primary Objective:

To obtain preliminary data on variability in PSMA expression by comparing prostate uptake on standard clinical ^{68}Ga-PSMA PET/CT with ^{68}Ga-PSMA PET/CT performed 4 weeks later.

Secondary Objective:

Dynamic imaging will be performed in order to get insight into the most ideal scan time.

3. STUDY DESIGN

This is a single centre prospective observation study without randomisation.

3.1 PSMA expression over time

The aim is to obtain preliminary data on variability in PSMA receptor expression by comparing prostate uptake on standard clinical ^{68}Ga-PSMA PET/CT with additional ^{68}Ga-PSMA PSMA PET/CT in a 4 week interval.

Primary prostate cancer patients will be included using routine MRI examinations. Based on protocol, a ^{68}Ga-PSMA PET/CT scan will be advised on high risk prostate cancer patients (cT3, Gleason score >7, PSA >20 ng/mL). These patients will be asked to participate in the study. If they agree to participate, the patients will undergo another ^{68}Ga-PSMA PET/CT scan within 4 weeks after the routine diagnostic scan. The protocol for the second scan is equal to the standard diagnostic ^{68}Ga-PSMA PET/CT scan. The PET scan will be acquired at approximately 45 minutes after administration of ~100MBq ^{68}Ga-PSMA.

In the 5 patients, a dynamic PET/CT-scan (dPET/CT) is acquired up to 40 minutes after administration of the radiopharmaceutical. The dPET/CT scan is performed at one bed position with the prostate in the central field-of-view (FOV). The low-dose CT scan of the same region is made before tracer injection, for attenuation correction and anatomic localization.[15] dPET/CT imaging is started immediately after intravenous injection of 100 MBq ^{68}Ga-PSMA.

The static PET/CT acquisition is performed directly after the dynamic series. Between the dynamic and static acquisition, the patient is requested to void.

Table 1. Division of subgroups in the clinical phase.

Phase	Subgroup	Administration for PET (MBq)
1a – dynamic and static	I. n = 5	100
1b – static	II. n = 25	100

4. STUDY POPULATION

4.1 Population (base)

30 patients with a primary prostate carcinoma will be included for this study. These patients should be eligible for a standard ^{68}Ga-PSMA -PET/CT-scan, according to clinical protocol. The patients will be recruited from the Urology department of the Antoni van Leeuwenhoek (AvL) hospital in Amsterdam. Initially, 30 patients, of whom 5 patients will undergo both dynamic and static PET/CT imaging, will be included. If a patient does not show PSMA expression on the first PET scan, this patient is excluded for the second scan, and will be replaced by another patient.

4.2 Inclusion criteria

A subject must meet all of the following criteria:

- Proven primary prostate carcinoma
- >18 years
- Good knowledge of Dutch language
- Written informed consent
- Underwent a pelvic MRI-scan
- Eligible for ^{68}Ga-PSMA PET/CT
- Lesion bigger than 10mm, to minimize partial volume effects

4.3 Exclusion criteria

A potential subject who meets any of the following criteria will be excluded from participation in this study:

- Contraindications for a PET/CT scan.
- Therapy scheduled between scans
- No PSMA expression on first PET scan

4.4 Sample size calculation

Paired measurements of 30 patients will provide 80% to show the equivalence of the two tests (4 weeks before surgery and 1 day before surgery) assuming that accepted boundary of equivalence is symmetric and equals 30%. A variation more than 30% is considered clinically relevant, according to PERCIST criteria[16]. We expect that the second measurement will be different from the first measurement by no more than 10%.Coefficient of variation is assumed to be between 0.6 and 0.7 and within-subject correlation is assumed to be 0.8-0.85.

5. NON-INVESTIGATIONAL PRODUCT

5.1 Name and description of non-investigational product(s)

^{68}Ga-PSMA contains PSMA which binds to the PSMA receptors. This is labelled with ^{68}Ga through an advanced radiolabelling process.

5.2 Summary of findings from non-clinical studies

Not applicable, the product is used as in usual clinical practice.

5.3 Summary of findings from clinical studies

Not applicable, the product is used as in usual clinical practice.

5.4 Summary of known and potential risks and benefits

5.5 Description and justification of route of administration and dosage

Not applicable, the product is used as in usual clinical practice.

5.6 Dosages, dosage modifications and method of administration

Patients will receive a venous catheter through which ±100 MBq of ^{68}Ga-PSMA is administered (maximum 10mL). The syringe is then flushed with 10mL of saline and removed. No dosage modifications are performed.

5.7 Preparation and labelling of Non-Investigational Medicinal Product

Preparation and labelling is performed according to standard protocol and current GMP-Z guidelines at the NKI-AVL.

5.8 Drug accountability

Not applicable, the product is used as in usual clinical practice.

6. METHODS

6.1 Study parameters/endpoints

The main study endpoint is to evaluate the PSMA expression variation at 4 week intervals, in terms of uptake difference in prostate and surrounding tissue). The secondary endpoint is to determine the optimal time between ^{68}Ga-PSMA injection and prostate surgery for future image-guided surgery using Cherenkov Light Imaging. Randomisation, blinding and treatment allocation

No randomization will take place. Scans will be anonymized. Each patient will be assigned a consecutive number. The first and the second scan will be assigned the letter ‘A’ or ‘B’, respectively. Only the researchers will be able to see the key, which allocates study numbers to patients. A nuclear medicine physician will assess the anonymized ^{68}Ga-PSMA PET/CT scans for differences in uptake. This physician is blinded to the order and correspondence between scans.

6.2 Study procedures

Phase 1a: dPET/CT + static PET/CT (n=5)
Phase 1b: static PET (n=25, total=30)

A schedule for one patient is as follows:
First scan ^{68}Ga-PSMA PET/CT scan as planned (standard clinical care).
Second scan~4 weeks later: ^{68}Ga-PSMA PET/CT scan (additional for research purposes).
The ^{68}Ga-PSMA PET/CT scan will be performed according to standard protocol. Patients will receive a venous catheter through which ±100 MBq of ^{68}Ga-PSMA is administered (maximum 10 mL). The catheter is then flushed with 10 ml of saline and removed. After 45 minutes a PET scan is performed directly followed by a low-dose CT scan. When the scans are finished, the patient can leave the hospital. The total procedure (including injection, resting, and scanning) lasts 1.5 hours.
Five patients will undergo a dPET/CT scan as well as a static PET/CT scan. To perform a dPET/CT scan, first a CT scan of the pelvis is made to ensure the location of the prostate. Thereafter, the patient will be injected with ^{68}Ga-PSMA while lying in the PET/CT scanner. Accordingly, the patient is continuously imaged for 40 minutes. After the scan, the patient is requested to void to better visualize the prostate. Then the standard static PET/CT scan is performed. This study does not interfere with standard clinical care. Both treatment and diagnostic procedures will not be altered or postponed for this study.

6.3 Withdrawal of individual subjects

Subjects can leave the study at any time for any reason if they wish to do so without any consequences. The investigator can decide to withdraw a subject from the study for urgent medical reasons. The investigator can decide to withdraw a subject from the study if on the first scan there is no PSMA expression visible. This can occur in 3% of the population. In this case, a second scan would not be useful as well.

6.4 Replacement of individual subjects after withdrawal

In case a subject does withdraw from the study or has no PSMA expression on the first PET scan a replacement is sought.

6.5 Follow-up of subjects withdrawn from treatment

Not applicable.

6.6 Premature termination of the study

Reasons for premature termination of the study are:

- Occurrence of any serious adverse events that can in anyway be related to the study.

7. STATISTICAL ANALYSIS

7.1 Primary study parameter(s)

All the data will be collected and quantitatively analysed within the NKI-AvL. The number and location of lesions are determined by a nuclear medicine physician. The absolute uptake in Bq/mL of the voxels will be determined using standard software (Osirix MD viewer). A paired t-test is used to compare uptake values of ^{68}Ga-PSMA of the first and the second scan.

7.2 Secondary study parameter(s)

The Bq/mL_{peak}, derived from the PET scan, and the size, derived from the low-dose CT scan, are determined of each lesion. These values will be compared between both scans.

7.3 Interim analysis (if applicable)

Not applicable.

8. ETHICAL CONSIDERATIONS

8.1 Regulation statement

The study will be conducted according to the principles of the Declaration of Helsinki and in accordance with the Medical Research Involving Human Subjects Act (WMO).

8.2 Recruitment and consent

A nurse practitioner, urology surgeon, oncologist or researcher will recruit patients in the out-patient clinic. Here, the subjects are informed about their clinical work-up and, if the subjects are eligible for inclusion, information about the study will be given both orally and in writing. Informed consent will be obtained at least a day prior to the PET/CT scan.
Find in the attachment the Patient Information and informed consent forms.

8.3 Objection by minors or incapacitated subjects (if applicable)

Not applicable

8.4 Benefits and risks assessment, group relatedness

This study does not directly benefit the patients included in this study. The risks associated with this study are low; the only extra procedure is an extra ^{68}Ga-PET/CT scan. The extra radiation burden from the administration of ^{68}Ga-PSMA is approximately 2mSv and ±4mSv from the low-dose CT-scan.[3] The pelvic low dose CT before the dPET/CT is approximately 1.5mSv. For the static group the total additional dose of this procedure is ±6mSv. For the dynamic group the additional dose is ±9mSv. This radiation burden is in the range of standard diagnostic tools and is not considered an additional clinically relevant risk in relation to their disease.

8.5 Compensation for injury

The sponsor/investigator has a liability insurance which is in accordance with article 7 of the WMO.

The sponsor (also) has an insurance which is in accordance with the legal requirements in the Netherlands (Article 7 WMO). This insurance provides cover for damage to research subjects through injury or death caused by the study.

The insurance applies to the damage that becomes apparent during the study or within 4 years after the end of the study.

9. ADMINISTRATIVE ASPECTS, MONITORING AND PUBLICATION

9.1 Handling and storage of data and documents

The acquired scans are handled and stored following the standard procedure as all scans in the hospital. Additional information about the patient is saved on local computers and anonymised. Patients are coded by a code consisting of a number, PET01, and each PET-scan is randomly designated with either_A or B. Number will be according to inclusion.. Only the involved researchers have access to the patient code. The encrypted data and the patient encrypted code key will not be stored on the same digital location. Data will be stored on the :S drive, only involved researchers will gain access through this drive. Data will be stored for the next 15 years, as described in the WGBO (Wet op de geneeskundige behandelingsovereenkomst).

10. STRUCTURED RISK ANALYSIS

10.1 Potential issues of concern

The results of this study can contribute to better understanding of the variation within ^{68}Ga-PSMA PET. This study does not directly benefit the patients included in this study. The risks associated with this study are low; the only extra procedure is an extra ^{68}Ga-PET/CT scan. The extra radiation burden from the administration of ^{68}Ga-PSMA is approximately 2mSv and ±4mSv from the low-dose CT-scan.[3] The pelvic low dose CT before the dPET/CT is approximately 1.5mSv. For the static group the total additional dose of this procedure is ±6mSv. For the dynamic group the additional dose is ±9mSv. This radiation burden is in the range of standard diagnostic tools and is not considered an additional clinically relevant risk in relation to their disease.

Since an additional PET/CT-scan is made, it requires inserting an additional venous cannula for administration of the ^{68}Ga-PSMA. An additional i.v. has minimal risk.

10.2 Synthesis

^{68}Ga-PSMA is used within the indication and administered according to standard clinical protocol. Therefore chapter 13.1 is skipped.

11. REFERENCES

1. Ferlay J, Soerjomataram I, Dikshit R, et al. Cancer incidence and mortality worldwide: Sources, methods and major patterns in GLOBOCAN 2012. *Int J Cancer*. 2015;136(5):E359-E386. doi:10.1002/ijc.29210.
2. Netherlands Cancer Registry. Managed by IKNL, March 2018. www.cijfersoverkanker.nl.
3. Evans JD, Jethwa KR, Ost P, et al. Prostate cancer–specific PET radiotracers: A review on the clinical utility in recurrent disease. *Pract Radiat Oncol*. 2018;8(1):28-39. doi:10.1016/j.prro.2017.07.011.
4. Minner S, Wittmer C, Graefen M, et al. High level PSMA expression is associated with early psa recurrence in surgically treated prostate cancer. *Prostate*. 2011;71(3):281-288. doi:10.1002/pros.21241.
5. Mafeld S, Vasdev N, Patel A, et al. Evolving role of positron emission tomography (PET) in urological malignancy. *BJU Int*. 2015;116(4):538-545. doi:10.1111/bju.12988.
6. Maurer T, Weirich G, Schottelius M, et al. Prostate-specific Membrane Antigen-radioguided Surgery for Metastatic Lymph Nodes in Prostate Cancer. *Eur Urol*. 2015;68(3):530-534. doi:10.1016/j.eururo.2015.04.034.
7. Demirkol MO, Acar Ö, Uçar B, Ramazanoğlu SR, Sağlıcan Y, Esen T. Prostate-specific membrane antigen-based imaging in prostate cancer: Impact on clinical decision making process. *Prostate*. 2015;75(7):748-757. doi:10.1002/pros.22956.
8. Eder M, Neels O, Müller M, et al. Novel preclinical and radiopharmaceutical aspects of [68Ga]Ga-PSMA-HBED-CC: A new PET tracer for imaging of prostate cancer. *Pharmaceuticals*. 2014;7(7):779-796. doi:10.3390/ph7070779.
9. Afshar-Oromieh A, Avtzi E, Giesel FL, et al. The diagnostic value of PET/CT imaging with the 68Ga-labelled PSMA ligand HBED-CC in the diagnosis of recurrent prostate cancer. *Eur J Nucl Med Mol Imaging*. 2014;42(2):197-209. doi:10.1007/s00259-014-2949-6.
10. Eiber M, Maurer T, Souvatzoglou M, et al. Evaluation of Hybrid 68Ga-PSMA Ligand PET/CT in 248 Patients with Biochemical Recurrence After Radical Prostatectomy. *J Nucl Med*. 2015;56(5):668-674. doi:10.2967/jnumed.115.154153.
11. Maurer T, Gschwend JE, Rauscher I, et al. Diagnostic efficacy of68Gallium-PSMA positron emission tomography compared to conventional imaging for lymph node staging of 130 consecutive patients with intermediate to high risk prostate cancer. *J Urol*. 2016;195(5):1436-1442. doi:10.1016/j.juro.2015.12.025.
12. Rahbar K, Weckesser M, Huss S, et al. Correlation of Intraprostatic Tumor Extent with 68Ga-PSMA Distribution in Patients with Prostate Cancer. *J Nucl Med*. 2016;57(4):563-567. doi:10.2967/jnumed.115.169243.
13. Lütje S, Heskamp S, Cornelissen AS, et al. PSMA ligands for radionuclide imaging and therapy of prostate cancer: Clinical status. *Theranostics*. 2015;5(12):1388-1401.

doi:10.7150/thno.13348.

14. Lückerath K, Stuparu AD, Wei L, et al. Detection threshold and reproducibility of ^{68} Ga-PSMA11 PET/CT in a mouse model of prostate cancer. *J Nucl Med*. 2018:jnumed.118.207704. doi:10.2967/jnumed.118.207704.
15. Uprimny C, Kroiss AS, Decristoforo C, et al. Early dynamic imaging in68Ga- PSMA-11 PET/CT allows discrimination of urinary bladder activity and prostate cancer lesions. *Eur J Nucl Med Mol Imaging*. 2017;44(5):765-775. doi:10.1007/s00259-016-3578-z.
16. Eisenhauer EA, Therasse P, Bogaerts J, et al. New response evaluation criteria in solid tumours: Revised RECIST guideline (version 1.1). *Eur J Cancer*. 2009;45(2):228-247. doi:10.1016/j.ejca.2008.10.026.
